# Detection of alpha-synuclein seeding activity in tear fluid in patients with Parkinson’s disease

**DOI:** 10.1038/s41531-026-01282-2

**Published:** 2026-02-18

**Authors:** Sezgi Canaslan, Matthias Schmitz, Fabian Maass, Peter Hermann, Susana Da Silva Correia, Shuyu Zhang, Bastian Popper, Wiebke Möbius, Christoph van Riesen, Piero Parchi, Paul Lingor, Inga Zerr

**Affiliations:** 1 Department of Neurology, University Medical Center Göttingen, Göttingen, Germany; 2 Department of Neurology, Klinikum rechts der Isar of the Technical University of Munich, Munich, Germany; 3 Biomedical Center, Core Facility Animal Models, Medical Faculty, LMU Munich, Munich, Germany; 4 EM Core Unit, Department for Neurogenetics, Max Planck Institute for Experimental Medicine, Göttingen, Germany; 5 Department for Biomedical and Neuromotor Sciences (DiBiNeM), University of Bologna, Bologna, Italy; 6 IRCCS Istituto delle Scienze Neurologiche di Bologna, Bologna, Italy; 7 German Center for Neurodegenerative Diseases (DZNE), Munich, Germany; 8 Munich Cluster for Systems Neurology (SyNergy), Munich, Germany; 9 German Center for Neurodegenerative Diseases (DZNE), Göttingen, Germany

**Keywords:** Neurology, Neuroscience

## Abstract

Detection of alpha-synuclein (α-Syn) seeding activity in tear fluid (TF) offers a promising non-invasive biomarker for Parkinson’s disease (PD). α-Syn seeding amplification assay (αSynSAA) detected seeding activity in 67% of PD-TF, while non-synucleinopathy samples remained negative. Electron microscopy of seeding-positive end products revealed fibrillar structures morphologically consistent with results of αSynSAA. αSynSAA effectively distinguished PD from controls and prion diseases based on seeding activity in TF.

The novel paradigm-shifting technology of protein amplification assays is based on induced protein misfolding and is extremely sensitive^1^. The alpha-synuclein amplification assays (αSynSAA) have been successfully applied to brain and skin tissue and cerebrospinal fluid (CSF) samples in synucleinopathies^2,3^. The method can potentially be extended to other accessible matrices, such as saliva and blood, and was used to identify the underlying pathology even preclinically^4,5^. Olfactory mucosa and saliva have already yielded positive results by αSynSAA^6,7^. There is also data available on exosomes from blood containing misfolded proteins^8,9^. However, complex matrices such as blood (and potentially oral and nasal swabs, too) bear the intrinsic problem of containing reaction inhibitors and other technological problems, as it has been demonstrated for CSF^10^. Tear fluid (TF) is a largely cell- and contamination-free biofluid, which can be accessed non-invasively and with little discomfort for the patient. In addition, innervation of the lacrimal gland originates in the brainstem, a structure that is affected early in PD. We therefore selected TF as a testing platform for our experiments.

SAA has been applied to detect misfolded prion protein in TF in prion diseases and shows positive results before the onset of clinical disease with similar diagnostic accuracy as for CSF^11^. Alpha-Synuclein (α-Syn) is detectable in TF of synucleinopathies, with increased abundance in PD^12^. In addition, there is evidence that α-Syn oligomers are detectable in saliva by αSynSAA^7^. We applied this powerful technology to amplify α-Syn seeds in TF from patients with PD and the control group. Morphology of α-Syn fibrils after αSynSAA was analyzed by transmission electron microscopy (TEM) in CSF and TF-seeded reactions.

## Development of an αSynSAA for TF

To develop a non-invasive diagnostic test for PD, we subjected TF from PD patients and controls to αSynSAA analysis over a 110-h period. Clinical characteristics of the patients are shown in Table 1.

**Table 1:** Demographic and clinical characteristics of the cohorts

	Age	Gender (F, M)	Modified H&Y stage	UPDRS III	Disease duration
PD	71.2 ± 8.7	4;16	3.1 ± 0.9	34.8 ± 12.3	8.7 ± 6.6
Controls	69.0 ± 9.7	5;13	NA	NA	NA

**Fig. 1:**
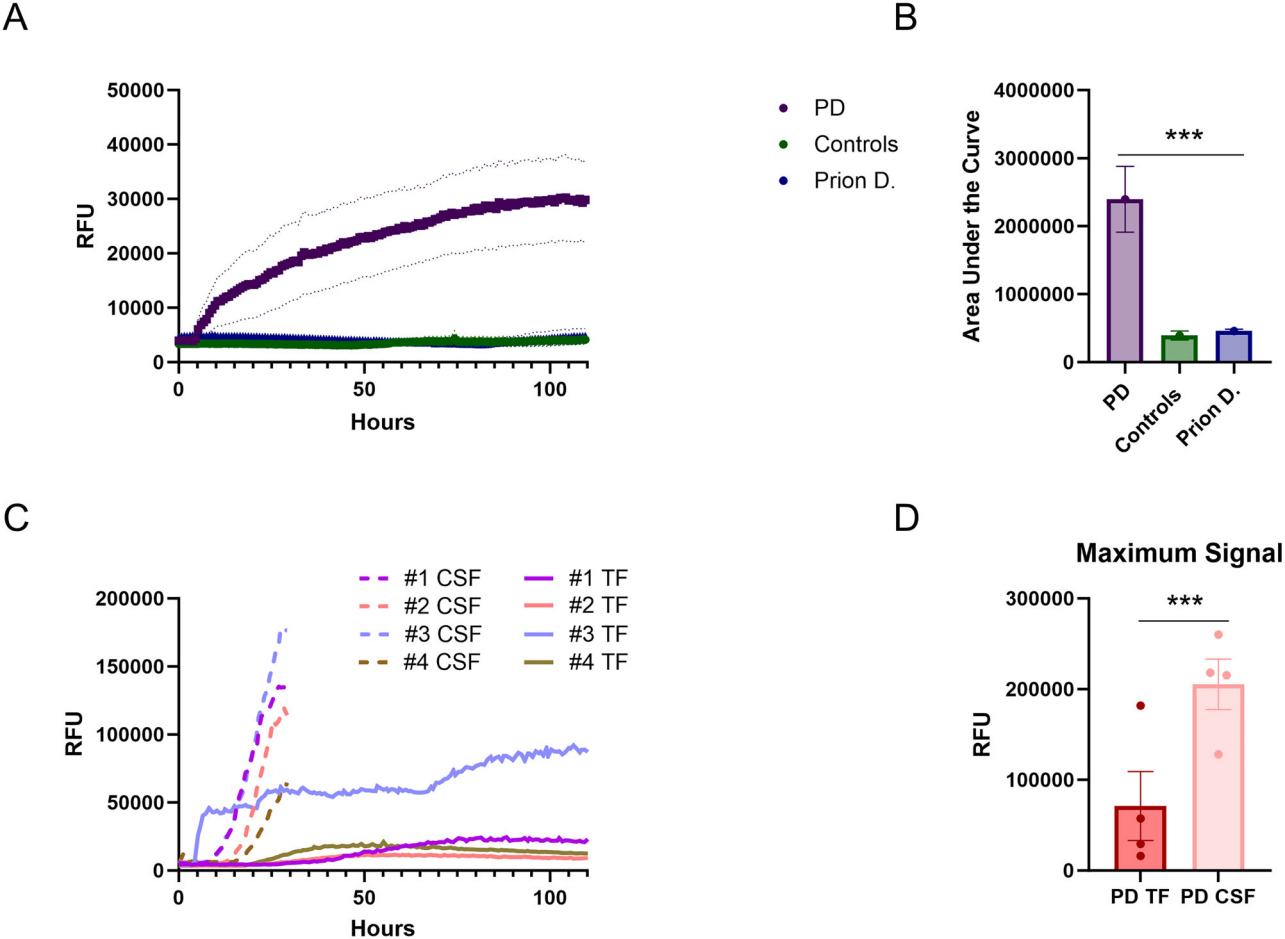
Comparison of α-Syn seeding performance in PD TF versus controls, and in PD TF versus CSF. **A** The solid lines show the overall mean of true positive samples (PD group: purple line), true negative samples (controls: green line), and prion disease samples (blue line). For convenience, we included PD samples with positive results and control samples with negative results (PD: *n* = 12, controls: *n* = 13, prion diseases: *n* = 5). Error bars indicate SEM. **B** Calculation of area under the curve values (AUC) indicates a significant difference between PD and controls (*p* < 0.001). **C**, **D** Samples (CSF and TF) from the same PD patients (*n* = 4) were analyzed in parallel, indicating that CSF-α-Syn produced a higher signal response in the αSynSAA assay than TF, as indicated by the signal maximum.

The kinetic curves were represented as the mean of all replicates from PD samples, which showed a marked signal response in TF (Fig. 1A). This significant increase in signal intensity suggests a strong seeding activity in PD samples, indicating the presence of pathological protein conformations associated with prion-like activity. In contrast, control samples from patients with prion diseases as well as non-neurological controls exhibited no detectable seeding activity (Fig. 1A). This lack of signal response was further supported by the significantly lower area under the curve (AUC) values observed for these control groups (Fig. 1B). The AUC values for both the prion disease patients and non-neurological controls were substantially reduced compared to the PD samples.

In our cohort of 21 patients with PD, seeding activity was detected in 67% of cases, with a specificity of 76% compared to controls and 83% compared to prion diseases (Supplementary Table 1). We adapted a recently suggested “two cutoffs” approach, which established the categories clearly negative, intermediate, and clearly positive. It has been applied in a number of clinical studies on CSF^13–15^. When only clearly negative and clearly positive cases were taken into account, sensitivity was 63% with excellent specificity (Supplementary Table 1).

## Comparison of α-Syn seeding activity in CSF and TF

We then aimed to compare the seeding activity of α-Syn in SAA reactions seeded with CSF and TF from the same patients. Data from 4 PD patients with matching pairs were available. Reactions were seeded with 15 µl of CSF or 15 µl of TF (Fig. 1C). The kinetic curves (average of a triplicate) of the matching CSF-TF samples from the same individuals are shown in (Fig. 1C); all of them were αSynSAA positive. The signal increase was much faster (shorter lag phase), and the maximal signal intensity was significantly higher in all CSF samples than in TF samples (Fig. 1D).

## Morphological characterization of α-Syn fibrils after αSynSAA by TEM

**Fig. 2:**
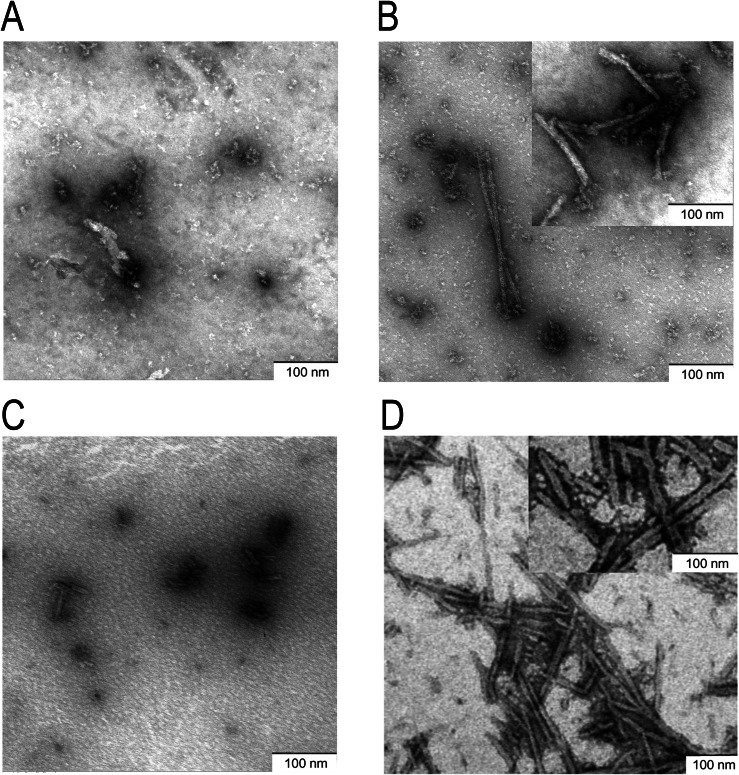
Morphological characterization of TF and CSF αSynSAA end products by transmission electron microscopy (TEM). Representative TEM images from αSynSAA reactions (*n* = 3 patients per group) after analysis. **A** Control TF-seeded reactions with a negative αSynSAA signal showed almost no fibrillar material. **B** Positive TF αSynSAA products seeded with PD revealed the formation of classical rod-shaped α-Syn fibrils (inset shows a magnification of fibrils). **C** CSF control-seeded reactions with a negative αSynSAA signal showed almost no fibrillar material. **D** Positive CSF αSynSAA products seeded with PD revealed the formation of classical rod-shaped α-Syn fibrils (inset shows a magnification of fibrils).

The morphology of αSynSAA products derived from TF and CSF was further characterized by TEM. Post-amplification, TEM demonstrated no significant fibrillar structures from αSynSAA negative controls (Fig. 2A, C).

The presence of extensive fibrils could be only found in reactions seeded either with PD TF or PD CSF samples (Fig. 2B, D). The amount and density of α-Syn fibrils are higher in reactions seeded with PD CSF than with PD TF (Fig. 2B, D). Magnificated fibrils (Fig. 2B, D insets) revealed marginal differences between CSF and TF-seeded αSynSAA reactions.

The α-Syn fibrils exhibited a rod-shaped morphology with variable size and compactness.

Prior αSynSAA no aggregated material is detectable, as demonstrated before^16^.

## Diagnostic accuracy of αSynSAA in TF compared to classical markers and CSF αSynSAA

For decades, research on biomarkers for neurodegenerative diseases was classically focused on CSF as a matrix because of its close relationship to the brain. In recent years, significant progress has been made concerning the development of blood-based assays, specifically in the field of Alzheimer’s disease and motor neuron diseases (Abeta, Tau, p-Tau, NFL)^17^. The αSynSAA technology goes even beyond and allows the detection of misfolded proteins in saliva and serum (in PD) and even in TF (in prion disease)^7^^,18,19,11^. Meanwhile, the technique has already entered the field of clinical diagnosis in prion diseases. CSF SAA has been highlighted as one of the best diagnostic strategies for synucleinopathies^20–22^. Moreover, it has already been proven effective for qualitative α-Syn detection in the CSF at the preclinical and early clinical disease stage^3–5,23,24^, and biological definitions and staging systems of PD based on the presence of α-Syn pathology have been suggested^21,22^.

Although CSF is the established biofluid for neurodegenerative diseases, the use of TF as a matrix for biomarker analysis has multiple advantages over other body fluids. The collection is non-invasive and can be performed repeatedly, even by trained non-medical personnel. It is safe and can be applied in a non-hospital setting, and it might therefore be very suitable for screening and monitoring purposes. It is free of contamination by blood and blood particles, which frequently occur in CSF samples and may obscure the results. The composition of TF might be even more suitable than CSF, because intrinsic inhibitors in CSF might affect the test results^10^.

Testing TF offers a unique opportunity for biomarker development, specifically for emerging technologies that are based on aggregation assays. Unlike surrogate biomarkers commonly proposed for many neurodegenerative disorders, which lack specificity, aggregation assays are based on the structural characteristics of the misfolded protein. These altered aggregation characteristics of the disease-related misfolded protein are used to amplify the seeds of the misfolded proteins of interest in a prion-like manner in vitro, followed by detection of the newly formed aggregated species using αSynSAA. This approach identifies tiny amounts of the misfolded species at the femtomolar level and is ideally suited to detect α-Syn aggregation activity in TF.

In our study, we demonstrated α-Syn seeding activity in TF derived from PD patients via αSynSAA. Using two independent cohorts, a discovery cohort and a validation cohort, we observed a positive signal in at least 67% of patients with PD. After exclusion of intermediate results, as it has been suggested for CSF^13,15^, we observed almost the same sensitivity to previous analysis (63%), but achieved a substantial improvement in specificity; none of our controls tested positive then. In clinical practice, we consider the high specificity of 100% as a convincing argument to prefer condition 2. When at least one replicate is positive, we recommend classifying the sample as being in a gray zone and, if sufficient material remains, we suggest repeating the analysis at least one time.

When comparing αSynSAA kinetics between CSF and TF from the same patients, we observed that CSF-seeded reactions have a shorter lag phase and higher signal intensity. One explanation is the higher concentration of α-Syn aggregates in CSF, providing more substrate for seeding. Alternatively, differences in α-Syn species in CSF and TF could lead to distinct aggregation behaviors. CSF typically contains higher levels of misfolded proteins like α-Syn, tau, and prion protein scrapie, which efficiently seed aggregation in the αSynSAA assay^12,20^. This results in more filament formation, stronger fluorescence, and faster kinetics compared to TF, where protein concentrations are much lower (α-Syn in CSF ranges from 100–300 pg/ml vs. 20–100 pg/ml in TF)^25^.

Another possible explanation is the differential ThT binding affinity to α-Syn fibrils with distinct structural properties. TEM analysis of αSynSAA end products revealed subtle morphological differences between fibrils seeded with CSF and those seeded with TF. Notably, reactions seeded with PD CSF yielded a higher abundance and greater fibril density than those seeded with PD TF. These findings suggest that CSF-derived α-Syn filaments may adopt a more ordered or compact conformation, which could enhance ThT binding, accelerate fibril growth, and ultimately result in increased seeding activity.

Our study is the first study to show seeding activity in TF in PD patients. Here, we used a similar approach as it has been used for human prion diseases by our group before^11^^,26^. Due to the nature of the first report, this study has some limitations. Samples were derived from one center only, and therefore, confirmatory analyses including samples from multiple centers are necessary. In CSF, a pooled sensitivity and specificity to differentiate synucleinopathies from controls with αSynSAA is reported with 0.88 (95% CI, 0.82–0.93) and 0.95 (95% CI, 0.92–0.97)^2^. The TF αSynSAA assay still requires optimization and currently exhibits lower sensitivity than CSF measurements; however, it offers a significant advantage over CSF αSynSAA due to its non-invasive nature and can serve as a valuable screening test prior to lumbar puncture. Correlative analyses with corresponding CSF samples in larger cohorts, including subgroups of PD patients with different clinical characteristics (early vs. late onset, tremor vs. bradykinetic onset, PD with and without RBD, etc.), are subject to further investigation in addition to atypical Parkinsonian syndromes. While our TEM analysis revealed the fibrillar morphology of the aggregates generated by αSynSAA, the molecular composition of these structures remains to be clarified. To further characterize the TEM structures, immunogold TEM could be performed to assess whether the aggregates are composed solely of α-Syn or contain additional proteins.

The development of an easily applicable assay in TF would represent an important milestone. Since protein aggregation begins years before the onset of motor symptoms, the timely identification of α-Syn accumulation is crucial for diagnostic purposes and may also allow early evaluation of clinical interventions. In particular, individuals in prodromal or early clinical stages are promising candidates for such interventions, underscoring the importance of establishing reliable diagnostic tools for disease detection at the earliest possible stage.

## Methods

### Patients

TF samples were obtained from the Movement Disorder Biobank at the Department of Neurology, University Hospital, Goettingen, Germany. Patient samples were collected to facilitate prospective research projects related to movement disorders. Parkinson’s disease patients were diagnosed according to the Movement Disorder Society criteria^27^. Control subjects with comparable age and sex, without clinical signs of neurodegeneration, were also selected from the biobank and consisted mostly of patients with peripheral neurological disorders like polyneuropathy or muscular disorders, plus one patient with subcortical arteriosclerotic encephalopathy. The patients underwent neurological examination, and a history was taken by movement disorder specialists. Patients were eligible regardless of disease duration or severity. Ophthalmological comorbidities and topical ocular medications were recorded. Only those with topical hyaluron administration were allowed. The diagnoses of prion disease cases were based on clinical criteria and *PRNP* genetic testing. CSF samples were obtained from the Movement Biobank, the prospective clinical study on cognitive decline in Parkinson’s disease (PARKA), and the Neurochemistry Lab of the Department of Neurology.

### Ethic approvals

Approval from local ethics committees was obtained (Ethics Committee of the University Medical Centre Goettingen, no. 37/11/21). Control samples from patients with prion disease were selected from the biobank of the German National Reference Center for Transmissible Spongiform Encephalopathies and donated TF as part of a surveillance and biomarker discovery study (Ethics Committee of the University Medical Centre Goettingen, no. 11/11/93).

All studies comply with the Code of Ethics of the World Medical Association (Declaration of Helsinki).

### TF collection

Tear collection was performed as previously described^12^ during the last 3 years. Briefly, tear samples were collected from both eyes using Schirmer test strips (Optitech, Allahabad, India). The strips were placed on the lower lid margin for 8 min and the wetting length of 10–20 mm (~5–10 µl per eye) was noted for each side. Patients with corneal inflammation or corneal ulcers were excluded. No topical anesthesia was used for sample collection. Samples were frozen immediately after collection and stored in polypropylene tubes at −80 °C until further analysis.

### Extraction of TF

For the extraction of TF from strips, TF extraction buffer was prepared in a final concentration of 40 mM phosphate buffer (PB), pH 8.0, 170 mM NaCl, and 0.0015% SDS. According to the wetting length of the strip, the required buffer volume was calculated as previously reported^11^ (15 mm requires 50 microliters TF extraction buffer). If the wetting length exceeded 15–20 mm, the strip was cut into 2–3 pieces depending on the total length using sterilized scissors, which were cleaned with 70% ethanol between each use to prevent contamination. After incubation on the shaker at room temperature for 1 h, strips were transferred to 0.5 ml Eppendorf tubes, from which the bottom part was cut. These tubes were inserted into 1.5 or 2 ml Eppendorf tubes to collect the TF. Samples were centrifuged at 13,000 rpm for 20 min. The extracted TF was collected and stored at −80 °C.

### αSynSAA protocol

The αSynSAA reactions are conducted following the established protocol outlined by Groveman et al., with minor changes^28^. The His-tagged α-Syn protein was purified using His-affinity chromatography and, later on, anion exchange chromatography as stated in Groveman et al.’s work with some additional changes^28^. The assay was performed on black 96-well plates with clear bottoms. Initially, each well was loaded with 6 × 0.8 mm molecular biology grade silica beads using a bead dispenser. The lyophilized recombinant α-Syn is resuspended in 520 ml of 40 mM PB, pH 8.0, and resuspension is filtered using a 100 kDa Amicon filter for 6 min at 10,000 rpm to remove potential oligomeric α-Syn. For the reaction mix, 85 μl were prepared per well, containing a final concentration of 40 mM PB pH 8.0, 170 mM NaCl, 10 µM Thioflavin-T, 0.0015% SDS, and 0.1 mg/ml of recombinant α-Syn in 100 μl. After adding 85 μl of the reaction mix to each well, 15 μl of the extracted TF sample or CSF was pipetted into the wells. Samples are tested in triplicates except for six samples, which either showed a complete positive or a complete negative outcome. The plate was sealed with a transparent plate sealer and placed in a Fluostar Omega plate reader. The incubation was carried out at 42 °C with double orbital shaking at 400 rpm for 1 min, followed by a 1-min rest period. Fluorescent measurements were taken every 45 min using 450 nm excitation and 480 emission filters for up to 30 or 110 h for CSF and TF, respectively. The run was considered positive when the fluorescence intensity reached at least two-fold of its initial value, measured 45 min after the start of the assay for TF and at least three-fold for CSF.

For the αSynSAA TF assay, we only used strips with a wetting length of more than 10 mm (preferably 15 mm), corresponding to approximately 10 µl of TF. Strips with shorter wetting lengths were excluded from the analysis because they did not provide sufficient sample volume to reliably perform the assay. Inconclusive samples were not repeated because the wetting lengths mostly provided sufficient material for a single assay run.

Each sample was analyzed in triplicates. The two-cutoff approach was applied. Samples performed 1 positive out of 3 replicates, considered intermediate or inconclusive. The data were analyzed in two cohorts. Additional analyses were performed after exclusion of inconclusive results (if only 1 out of 3 runs were positive). The first cohort comprised samples from 12 patients with PD and 12 neurological non-neurodegenerative controls. After obtaining encouraging results, the second cohort was tested, which comprises nine PD patients, five neurological non-neurodegenerative controls, and six prion disease patients, with similar results as in cohort 1. The analyses were done by combining two cohorts to have more meaningful results.

### Transmission electron microscopy (TEM)

TEM was performed to examine the solutions with 300 µM fibrillized αSyn after αSynSAA. A Formvar-coated copper EM-grid was floated on 10 µl of sample, followed by the addition of 10 µl of 0.25% glutaraldehyde. After 1 min, the grid was washed in three drops of water. For contrast, the grid was incubated with 2% aqueous uranyl acetate solution for 30–60 s. Excess uranyl acetate solution was removed by gently touching the grid vertically with a piece of filter paper. The negative-stained samples were imaged with TEM, and the digital micrographs were obtained with an on-axis 2048 × 2048-CCD camera (TRS, Moorenweis, Germany).

## Supplementary information

Supplementary Table 1

## Data Availability

The datasets generated and/or analyzed during the current study are not publicly available due to ongoing research that relies on the data, but are available from the corresponding author on reasonable request.
