# Supplementary material for: Detection of alpha-synuclein seeding activity in tear fluid in patients with Parkinson’s disease
Source: NPJ Parkinsons Dis. 2026 Feb 18;12:66. doi: 10.1038/s41531-026-01282-2 (PMC12992906; doi:10.1038/s41531-026-01282-2)
Supplement: Supplementary file 1 — Supplementary Table 1 [file 41531_2026_1282_MOESM1_ESM.docx]

**Suppl. Table 1: Diagnostic accuracy of the aSynSAA assuming two different conditions when at least one reactions is positive (condition 1) and after exclusion of intermediate results (condition 2).**

**Positivity 0/3 1/3 2/3 3/3 Total (n) Sensitivity Specificity**

| **Condition 1: At least 1 sample is positive** | | | | | | | |  |
| --- | --- | --- | --- | --- | --- | --- | --- | --- |
| **PD** | 7 | 2 | 0 | 12 | 21 | 67% |  | |
| **Controls** | 13 | 4 | 0 | 0 | 17 |  | 76% | |
| **CJD** | 5 | 1 | 0 | 0 | 6 |  | 83% | |
|  | **Condition 2: Exclusion of intermediate results** | | | | | | | |
| **PD** | 7 |  |  | 12 | 19 | 63% |  | |
| **Controls** | 13 |  |  | 0 | 13 |  | 100% | |
| **CJD** | 5 |  |  | 0 | 5 |  | 100% | |
